# Impact of introducing a capacity-based mental health law in Norway: qualitative exploration of multi-stakeholder perspectives

**DOI:** 10.1192/bjo.2024.810

**Published:** 2025-02-25

**Authors:** Jacob Jorem, Reidun Førde, Tonje Lossius Husum, Jørgen Dahlberg, Reidar Pedersen

**Affiliations:** Institute of Health and Society, University of Oslo, Oslo, Norway; Department of Health Care Policy, Harvard Medical School, Boston, USA; and Department of Health Policy and Management, Columbia University Mailman School of Public Health, New York, USA; Institute of Health and Society, University of Oslo, Oslo, Norway; Faculty of Health Sciences, Oslo Metropolitan University, Oslo, Norway

**Keywords:** Consent and capacity, psychiatry and law, mental health services, qualitative research, ethics

## Abstract

**Background:**

Decision-making capacity (DMC) is key to capacity-based mental health laws. In 2017, Norway introduced a lack of DMC as an additional criterion for involuntary care and treatment to strengthen patient autonomy and reduce involuntary care. Health registry data reveal an initial reduction followed by rising involuntary care and treatment rates post-2017. Despite jurisdictions moving towards capacity-based mental health laws, little is known about their impact.

**Aims:**

To explore the impact of introducing a capacity-based mental health law governing involuntary care and treatment.

**Method:**

Semi-structured interviews and focus groups were conducted in 2018 with 60 purposively sampled stakeholders, including patients, families, health professionals and lawyers. Of these, 26 participated in individual follow-up interviews in 2022–23. The transcribed interviews were thematically analysed following Braun and Clarke.

**Results:**

Four themes emerged: (a) increased awareness of patient autonomy and improved patient involvement; (b) altered thresholds for involuntary admission and discharge and more challenging to help certain patient groups; (c) more responsibility for primary health services; and (d) increased family responsibility but unchanged involvement by health services.

**Conclusions:**

Introducing a capacity-based mental health law appears to raise awareness of patient autonomy, but its impact depends on an interplay of complex health, social and legal systems. Post-2017 changes, including rising involuntary care and treatment rates, higher thresholds for admissions and increased pressure on primary health services and families, may be influenced by several factors. These include implementation of decision-making capacity, legal interpretations, formal measures for care of non-resistant incompetent individuals, reduced in-patient bed availability, inadequate voluntary treatment options and societal developments. Further research is needed to better understand these changes and their causes.

Capacity-based mental health laws aim to uphold patient autonomy while ensuring the need for care.^1–3^ Decision-making capacity (DMC) is the key criterion of these laws and typically involves patients’ ability to understand, reason and appreciate relevant information and communicate a choice.^4–6^ The lack of DMC implies an inability to give valid consent to care or treatment.^7^ In addition to DMC, capacity-based mental health laws typically mandate procedural rights to safeguard patient autonomy.^8^ These laws are gaining international prominence in line with human rights developments, particularly the United Nations Convention of the Rights of Persons with Disabilities (CRPD).^1,7^

In 2017, a lack of DMC was introduced as an additional criterion for involuntary care and treatment as part of comprehensive amendments to the Norwegian Mental Health Care Act (MHCA) (see Appendix Table 3). The 2017 amendments also introduced additional procedural rights to ensure patient autonomy (see Appendix Table 4). Among the existing criteria for involuntary care were severe mental illness, need for treatment and danger to self or others. Importantly, the DMC criterion does not apply in cases of danger to the person's life or the life or health of others. Unless posing such a danger, competent individuals can no longer be involuntarily admitted or treated. However, both DMC and danger assessments can pose challenges in practice.^2,9,10^ Under Norwegian health law, physicians in primary or specialist care evaluate whether these criteria under the MHCA are met when referring patients for involuntary hospitalisation. Within 24 h of the patient’s admission, psychiatrists or specialists in clinical psychology make an initial decision regarding involuntary care. Follow-up options include continued hospitalisation or out-patient care under Community Treatment Orders (CTOs) (see Appendix Fig. 2).

In several high-income countries, rates of involuntary mental healthcare are rising despite various policies aimed at reducing these rates.^11^ The 2017 amendments aimed to strengthen patient autonomy while reducing both the rates and duration of involuntary care and treatment, particularly CTOs, by narrowing the scope of the existing need for treatment criterion. A 2023 report by an Expert Commission on DMC in mental healthcare analysed Norwegian national health registry data, revealing an initial reduction followed by increasing involuntary care and treatment rates, consistent with post-2017 trends.^10^ This amounted to a 10% increase per capita in involuntary admissions from 2016 to 2022 (see Appendix Fig. 3). During the same period, the number of people under CTOs rose by 17% per capita, and there was an almost threefold increase in involuntary treatment decisions.^10,12^ Moreover, increased proportions of acute and involuntary admissions suggest a shift towards people becoming more severely ill since 2017.^10^ Additionally, a rise in referred patients not being admitted to involuntary care since 2017 indicate an increased threshold for admissions.^10^ In parallel, the average length of hospitalisations has remained stable, and overall readmissions within 30 days after discharge have not significantly changed from 2016 to 2022. However, individuals with comorbid substance misuse have experienced more frequent readmissions and reduced hospitalisation durations after 2017.^10^

While health laws vary across jurisdictions, several are moving towards capacity-based mental health laws like those in the UK.^1,2,13^ Northern Ireland has enacted its Mental Capacity Act as a ‘fusion law’, combining both mental and physical health legislation. This act focuses on a lack of DMC to make treatment decisions irrespective of cause, but it has not yet been fully implemented.^14^ Despite movements towards implementing capacity-based mental health laws, there is limited empirical research on their impact. In this regard, the early implementation experiences in Norway offer valuable insights. This study aimed to explore the impact of introducing a capacity-based mental health law in Norway governing involuntary care and treatment.

## Method

This study used qualitative semi-structured interviews and focus groups conducted in 2018 and semi-structured interviews in 2022–23 with key stakeholder groups. These included patients, families, psychiatrists, specialists in clinical psychology, general practitioners and lawyers in supervisory bodies (Control Commission and County Governor). It was part of a larger research project at the Institute of Health and Society (IHS), University of Oslo, exploring various aspects of Norway’s introduction of DMC in 2017, including DMC assessments in clinical practice and factors influencing these assessments.

This study addressed the following research question: what kind of impact do patients, families, health professionals and supervisory bodies in Norway experience when a capacity-based mental health law governing involuntary care and treatment is introduced?

### Study design

This qualitative study conducted semi-structured interviews and focus groups in 2018 with 60 stakeholders, 26 of whom participated in individual follow-up interviews in 2022–23.

## Research group

The research group at IHS consisted of researchers (J.J., R.F., T.L.H., J.D., R.P.) with backgrounds in psychology, psychiatry, medicine, law and ethics. The research group was composed of three males and two females, all with prior knowledge of DMC and involuntary mental healthcare, as well as teaching experience in different healthcare settings. Given the small Norwegian mental healthcare community, participants may have been familiar with the interviewer. However, the group sought to avoid interviewers having established relationships with participants prior to starting this study. Moreover, no characteristics about the interviewer were shared before or during the interviews. The research group evaluated researchers’ influence on study validity throughout the process by engaging in reflexivity.^15^

### Setting

The individual interviews and focus groups took place in both urban and rural locations across Norway, primarily in participants’ offices or at another location of their choosing. One interview in 2022–23 was conducted digitally.

### Sampling and recruitment

Purposive sampling was used to identify and select participants experienced in DMC in mental healthcare in 2018.^16^ Participants with experience in involuntary mental healthcare and treatment, both before and after the 2017 amendments, were regarded as particularly relevant to explore the impact of introducing a capacity-based mental health law. Clinicians from various primary and specialist care roles were selected, including those working in general practice, urgent care clinics, psychiatric wards and out-patient clinics.

Due to known geographic variations in involuntary care rates across Norway, participants were sampled from geographical areas that reflected these variations based on national health registry data.^17,18^ Four geographic areas in different parts of Norway were selected: three urban and one rural. Participants from specialist and primary care were recruited from all four areas, while those from supervisory bodies, patients and families came from two areas due to recruitment challenges in the other two.

Using a reputational sampling approach, participants for the 2018 interviews were recruited through national organisations representing key stakeholders, who recommended experts on DMC across the four geographic areas. These recommended participants, in turn, suggested other participants with experience with DMC and involuntary care and treatment. In both interview rounds, participants were initially contacted via email, followed by phone calls. Data saturation was discussed throughout the recruitment process within the research group.^19^

A stakeholder advisory group of seven members was recruited as part of the larger research project at IHS through recommendations from national organisations representing patients, families, clinicians and lawyers. This advisory group met with the research group, providing valuable input and recommendations for each of the project’s studies. Participants from supervisory bodies were recruited based on recommendations from the advisory group. Patient and family participants were recruited through national organisations.

Participants from the 2018 interview were invited to take part in the 2022–23 individual follow-up interviews, aiming to reinterview as many as possible. However, clinical and supervisory body participants were required to have at least 2 years of relevant experience since the 2018 interviews to be eligible. This inclusion criterion did not apply to patient and family participants, all of whom were invited to take part in the follow-up interviews. While 26 of the same participants took part in 2022–23, reasons for non-participation among the remaining 34 participants included failing to meet the inclusion criterion of 2 years of relevant experience since 2018 (9), time constraints (7), health issues (3), retirement (3) and communication challenges (12).

### Individual interviews and focus groups

In 2018, 60 participants were interviewed, either individually (43) or in focus groups (17). Five separate focus groups were conducted to enhance the comfort of speaking freely and upon participants’ request. The groups included patient (4), family (5), specialist care (3), primary care (2) and supervisory body (3) participants. This approach aimed to minimise potential peer effects of differences in status and hierarchy on individual responses in groups. All patient participants in 2018 took part in focus groups, and none were individually interviewed. Individual interviews lasted approximately 60 min, while focus groups extended to 90 min. The same semi-structured interview guide was used consistently for both individual and group settings, allowing participants to share experiences, explain their reasoning and ask questions (see Supplementary Material 1 available at https://doi.org/10.1192/bjo.2024.810). The five focus groups were led by either T.L.H. or J.D. The 43 individual interviews were conducted by one of four researchers from IHS (T.L.H., R.P., J.D., J.J.), with two interviews conducted jointly by J.J. and R.P.

In 2022–23, individual follow-up interviews were conducted with 26 of the the same participants from the first phase of data collection, using the same interview guide as in 2018. Participants in 2022–23 were asked to reflect on changes since the last interview or focus group as part of their interview instructions, a researcher with a background in psychiatry and law (J.J.) conducted 23 interviews. J.J. jointly conducted two interviews with another researcher with expertise in medicine and ethics (R.P.), while R.P. conducted one interview independently. In 2022–23, half of the participants were female, and 65% were aged 50 or older. All participants from specialist and primary care had a minimum of 5 years of clinical experience in 2022–23, and 83% had 15 years or more of experience. Several of the interviewed health professionals held managerial positions and emergency medical responsibilities. Some of them worked across various departments, including urgent care centres, district psychiatric centres, geriatric psychiatry wards and secure units (see Table 1).

**Table 1:** Roles and number of participants in the 2018 individual interviews and focus groups and the 2022–23 individual follow-up interviews

	2018 interview and focus group		2022–23 interviews	
Role	Population (*N* = 60) (%)	Individual interviews (*N* = 43) (% of 2018 interviews)	Focus groups (*N* = 17) (% of 2018 interviews)	Population (*N* = 26) (%) (all individual)
Patient experience	4 (7)	0 (0)	4 (100)	1 (4)
Family experience	12 (20)	7 (58)	5 (42)	4 (15)
Specialist care^a^	27 (45)	24 (89)	3 (11)	16 (62)
Primary care^b^	12 (20)	10 (83)	2 (17)	2 (8)
Supervisory bodies^c^	5 (8)	2 (40)	3 (60)	3 (12)

a. Specialists in psychiatry and clinical psychology.

b. General practitioners, physicians at urgent care centres and district medical officers.

c. Control Commission (Kontrollkommisjonen) and County Governor (Statsforvalteren) (refer to Appendix Fig. 2 for clarification).

The interviews were audio-recorded and transcribed. Research assistants at IHS verified the correspondence between the audio recordings and transcripts under the guidance of the research group. The research group also took field notes during and after the individual interviews and focus groups to provide context for the data analysis..

This study analyses the responses of participants to the following questions in the interview guide (see Supplementary Material 1):

- What are your experiences with the impact of introducing DMC as part of the amendments to MHCA in 2017?
- How have the legislative amendments influenced your practice in other areas of health law?

### Data analysis

Preliminary analyses of the 2018 interviews and focus groups were conducted by four researchers (T.L.H., R.P., J.D., J.J.). After the 2022–23 interviews were conducted, J.J., R.P. and R.F. independently analysed the data from both rounds of interviews. The transcribed interviews were thematically analysed following Braun and Clarke.^15^ The analytic process began with J.J., R.P. and R.F. familiarising themselves with the audio recordings and transcripts from 2018 and 2022–23. The data content was sorted into initial codes, which were adjusted based on the coding of each research group member. This coded data was analysed to generate potential themes and subthemes, which were then refined to ensure their internal coherence and validity in relation to the entire data-set. The themes and subthemes were further refined and defined as well as the data within them reanalysed, concluding in the study write-up.^15,19–22^ All members of the research group contributed to coding and further analysing the data.^15,20^ NVivo (Windows Release 1.6.1) was used to facilitate data management. Changes over time from the first collection of data to the second were identified through both the reflection of participants in the 2022–23 interviews and a comparative analysis of the two rounds of interviews, with all transcripts being analysed together. Rather than comparing the responses of the same 26 participants in 2018 and 2022–23, all 60 participants in 2018 were included to ensure variation in perspectives. Preliminary findings and data analysis of this study were reviewed by the stakeholder advisory group, and their recommendations further informed the analytical process.

### Ethics

Participants received written information about the research project before the interviews. This information was reiterated verbally at the start of the interviews. Written informed consent was obtained from all participants. Findings are presented in a manner that ensures participant anonymity. Access to the data has been limited to personnel involved in the study, and all data are stored on the University of Oslo's services for sensitive data. The authors assert that all procedures contributing to this work comply with the ethical standards of the relevant national and institutional committees on human experimentation and with the Helsinki Declaration of 1975, as revised in 2013. All procedures involving human subjects were approved by the Regional Committee for Medical and Health Research Ethics (2018/488/REK South-East), the Data Protection Officer at the University of Oslo and the Norwegian Agency for Shared Services in Education and Research (Sikt (400975)).

## Results

Analysis of the 2018 and 2022–23 interview data generated four themes and seven subthemes (see Table 2). While stakeholder groups generally shared similar views, notable differences are highlighted in this section. The illustrative quotes are from the 2022–23 interviews, as participants had more experience with the impact of DMC and the broader legislative amendments. The 2018 interviews serve as a key reference point for exploring changes over time.

**Table 2:** Themes, subthemes and illustrative quotes from 2022–23 regarding the impact of introducing a capacity-based mental health law in Norway in 2017 (see Appendix Table 5 for additional quotes)

Theme	Subtheme	Quote (type of participant, participant number)
1. Increased awareness of patient autonomy and improved patient involvement	1A. Increased awareness	‘A positive aspect is that the legislative amendments have increased awareness and placed more focus on patients with severe mental illness. They have shifted our automatic assumption that these patients are incapable of managing their lives. […] There is a more nuanced approach in both assessments and attitudes. […] There is indeed a greater emphasis on autonomy and self-determination.’ (Psychiatrist, 1, Q1)
	1B. Improved patient involvement	‘As specialists [in psychiatry or clinical psychology], we have become more attentive to comprehensively assessing capacity. Patients have become better informed about their rights, and I have been somewhat more focused on patient choice.’ (Specialist in clinical psychology, 24, Q2)
2. Altered thresholds for involuntary admission and discharge and more challenging to help certain patient groups	2A. Increased admission threshold and reduced discharge threshold	‘The threshold for discharge might be lower due to the limited number of available beds. There is a need to accommodate more patients, resulting in faster discharges.’ (Family participant, 13, Q3)
	2B. More challenging to help certain patient groups	‘Some patients, particularly those with bipolar disorder, can rapidly regain capacity with the right medication. Upon regaining capacity, they can make their own decisions and discontinue medication.’ (Supervisory body participant, 4, Q4)
3. More responsibility for primary health services with inadequate assessment training		‘Primary health services are pressured to take care of severely ill patients. […] And not everyone in the specialist health services understands that primary health services do not have involuntary care measures available.’ (Supervisory body participant, 11, Q5)
4. Increased family responsibility but unchanged involvement by health services	4A. Increased responsibility	‘I believe it has been more burdensome for families since 2017.’ (Psychiatrist, 3, Q6)
	4B. Unchanged involvement by health services	'When it comes to involuntary care assessments, I believe families are still far too seldom involved. In a few cases, they were heavily involved, perhaps because they are strong advocates and easy to work with. However, in many cases, families are minimally involved. This not only concerns families themselves but also our access to information to understand how things really are. […] Also, not all families wish to be involved, particularly in cases where the same issues arise repeatedly.' (Psychiatrist, 10, Q7)

### Patient autonomy and involvement

#### Increased awareness of patient autonomy

‘The positive aspect of the legislative amendments perhaps lies in enhanced legal protection or strengthened autonomy for the patients, and in our increased awareness of it’ (Psychiatrist, 17, Q8).

Most participants stated that the capacity-based mental health law had increased their awareness of patient rights. According to some participants from specialist care and supervisory bodies, DMC had become an integral part of comprehensive clinical assessments for involuntary care and treatment decisions. They regarded DMC as both a clinical and legal criterion influencing patient interactions and making health professionals more conscious of the importance of voluntary treatment options. According to them, DMC contributed to improving the alliance between patients and health professionals. However, they viewed DMC as only one of several initiatives in recent years to raise awareness of patient autonomy. Moreover, a stronger emphasis on patient autonomy could lead to what they considered as inadequate treatment provision. They also highlighted that it would take time to achieve substantial reductions in involuntary care rates.

#### Improved patient involvement

‘When we talk about a more comprehensive assessment, it does not necessarily imply that it takes much longer, but rather that it results in a more accurate assessment of the patient’ (Primary care participant, 23, Q9).

Several participants described longer and more comprehensive conversations between health professionals and patients following the legislative amendments. This also involved asking about individuals’ perspectives on their own situation and treatment. Several specialist care participants noted increased acknowledgement of people's resources and capabilities following the introduction of DMC. They also highlighted health professionals being more attentive to involving patients in decision-making processes, and DMC assessments serving as a reminder of the significance of patient autonomy. According to these participants, they had become more aware of their rights related to voluntary treatment options after 2017. This contributed to more comprehensive assessments, where more emphasis was placed on people's rights to express their views on involuntary care and treatment before decisions were made (advanced statements) (see Appendix Table 4). These participants highlighted that they and their families also gained a better understanding of clinicians’ assessments and the reasoning behind treatment recommendations.

### Altered admission and discharge thresholds and challenges in helping patient groups

#### Increased admission thresholds and reduced discharge thresholds

‘Several individuals in need of treatment do not receive it. [ … ] Treatment is often initiated while capacity is lacking, only to be regained during therapy. Consequently, many discontinue treatment, experience a deterioration in their condition’ (Family participant, 12, Q10).

Specialist care participants observed that DMC made it more difficult to admit people until they became more severely ill, and harder to retain once capacity was regained. They noted that individuals in the grey area of DMC were more likely to go untreated following the legislative amendments. Additionally, the increased admission threshold posed a challenge to their preventive efforts, as people had to become sufficiently ill before providing what they considered necessary healthcare.

Some specialist care participants questioned whether it was the wording of the law and legal interpretations of DMC that prevented people from receiving such care. They emphasised that there was little room for doubt in DMC assessments. This was reflected in the altered statutory objectives in the MHCA for preventing and limiting the use of involuntary care and the strict evidentiary requirement of ‘obviously’ lacking DMC (see Appendix Table 3). According to some, this may have contributed to a higher threshold for involuntary admission and a lower discharge threshold. Moreover, some noted that the requirement to use formal involuntary care and treatment measures for individuals lacking DMC, even when they did not resist admission or treatment, contributed to the rising involuntary care and treatment rates after 2017. While family participants acknowledged the benefits of introducing DMC for people capable of managing their treatment, they highlighted that many competent participants and their families lacked the resources to do so.

#### More challenging to help certain patient groups

‘DMC concerns the most vulnerable patients, suffering from severe mental illness, often with comorbid substance misuse. I find that they frequently fall through the cracks because they regain capacity, shortening the duration of involuntary admission’ (Psychiatrist, 2, Q11).

Several participants voiced concerns that DMC made it more challenging to help certain patient groups. These included people with manic symptoms and comorbid substance misuse whose mental state, including DMC, often fluctuated. This made them particularly vulnerable to altered thresholds for both involuntary admission and discharge. Several participants, particularly in the 2022–23 interview round, perceived an increase in the frequency of readmissions following the legislative amendments. This increase was particularly notable among people with comorbid substance misuse who were either assessed as competent before admission or quickly regained DMC afterward. Consequently, they were discharged with the potential for continued substance misuse. Individuals with manic symptoms were also perceived as having similar clinical pathways, often being discharged after an appeal to the Control Commission, with a longer period before readmission. However, some participants noted that providing healthcare for manic symptoms and comorbid substance misuse had posed challenges even before the 2017 amendments. Moreover, some participants emphasised that certain developments in mental healthcare post-2017 appeared to be attributed to DMC, even though it may not have played a key role. These developments included an increased number of ‘revolving door’ patients.

### More responsibility for primary health services with inadequate assessment training

‘For primary health services, it is a significant dilemma to admit and discharge patients. [ … ] Providing quality services for those with fluctuating decision-making capacity is difficult. [ … ] It takes very little for it to collapse. Primary health services are not able to maintain stability for many of the sickest patients’ (Family participant, 12, Q12).

Several participants described increased responsibility for primary health services to follow up people with long-term care needs after 2017. According to family participants, this shift was partly related to the challenges of people being discharged earlier and higher thresholds for admission to specialist health services. Participants from primary and specialist care observed that primary health services were not adequately equipped to handle the increased number of competent individuals with severe mental illnesses (SMIs) receiving voluntary care who wanted neither specialist healthcare nor antipsychotic medication. While this shift required closer collaboration among different levels of the healthcare system, they had not experienced it. Moreover, several specialist care participants emphasised that the implementation of the legislative amendments had been inadequate, including a lack of systemic training and validated tools. This was particularly noticeable within primary health services. They highlighted that insufficient information about the legislative amendments and how to assess DMC led to uncertainty in primary care, adding to the challenges of introducing a capacity-based mental health law.

### Increased family responsibility but unchanged involvement by health services

#### Increased responsibility

‘When [ … ] they do not receive necessary healthcare, the burden on families becomes significantly greater. Family may call in a state of utter despair and appeal a decision to terminate involuntary admission and treatment. I have no doubt that the burden on families has increased substantially’ (Supervisory body participant, 6, Q13).

Several participants described increased family responsibility since 2017 because of greater difficulties in accessing recommended treatment. Consequently, families found themselves caring for people who are more severely ill who previously may have been under involuntary care and treatment. Some participants attributed this increased burden to resource constraints in mental healthcare, including reduced availability of in-patient beds and limited out-patient follow-up. Some specialist care participants highlighted that families of individuals with substance-induced psychoses were particularly vulnerable after 2017 because of their fluctuations in DMC. They highlighted the fact that most families were supportive of individuals being hospitalised. According to them, the altered thresholds for admission and discharge increased family responsibility when competent individuals refused care. This included ensuring that participants adhered to their medication and contacted the health services if their condition deteriorated. Family participants emphasised that their responsibility also increased when there was a lack of cooperation between the specialist and primary health services.

#### Unchanged family involvement by health services

‘The wishes and experiences of the families are not considered to a significant extent. It is the clinical conversation and our assessment of the patient that is the primary focus’ (Psychiatrist, 2, Q14).

Despite the increased family responsibility, several participants observed that there had not been a corresponding increase in their involvement by health services. Specialist care participants highlighted insufficient family involvement both during involuntary admissions and CTOs, and after discharge from health services. Some of them believed that the legislative amendments had not changed their approach to family involvement. Others emphasised an increased focus on early family involvement, especially for newly diagnosed patients. The level of family involvement in DMC assessments varied according to family and supervisory body participants. However, family participants found that the degree of family involvement, including in DMC assessments, largely depended on their own efforts to become involved. Some specialist care participants expressed uncertainty about handling confidentiality when interacting with families, citing complex confidentiality laws and professional ethical norms as barriers to involving families, regardless of the individual's DMC status.

## Discussion

### Summary of main findings

Several changes in mental healthcare were described after introducing a capacity-based mental health law in Norway in 2017. The legislative amendments seem to have contributed to raising awareness of patient autonomy and improved patient involvement. Participants also reported higher thresholds for admission and lower thresholds for discharge post-2017, along with people who are more severely ill in both in-patient and out-patient settings. Reasons for these changes varied among participants, including altered statutory objectives to prevent and limit involuntary care and treatment rates, the strict evidentiary requirement of ‘obviously’ lacking DMC (see Appendix Table 3), inadequate implementation of DMC and reduced availability of in-patient beds.^10^ Moreover, participants described increased responsibility for primary health services and families in following up people with SMIs. Despite these changes, collaboration between health service levels, family involvement and emphasis on voluntary healthcare had not improved, according to participants.

### Contextual factors

**Fig. 1:**
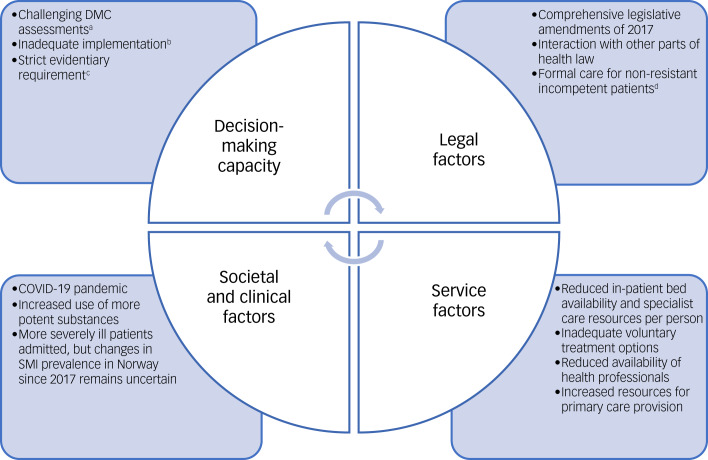
The experiences with decision-making capacity (DMC) since 2017 must be viewed in conjunction with broader factors, including legal and health service, as well as societal and clinical. ^a^Some studies suggest that DMC assessments can be challenging in practice.^2,9,28^ ^b^The inadequate implementation of DMC in Norway and the need for quality assurance measures for assessments include systematic training and validated tools.^10^ ^c^A strict evidentiary requirement of ‘obviously’ lacking DMC leaves little room for doubt in DMC assessments (see Appendix Table 3). ^d^An existing requirement in Norwegian health law mandates the use of formal involuntary care measures when incompetent patients do not resist care or treatment (see Appendix Table 4).

These findings can be seen in relation to other changes in Norwegian health services and society since 2017 (see Fig. 1), some of which are also relevant internationally. Service factors include reduced in-patient bed availability in mental healthcare, with a 30% reduction per capita in Norway between 2011 and 2021.^10^ In recent years, there has also been an increased number of people admitted to these civil psychiatric wards on criminal grounds.^10^ Less access to early interventions, resulting from factors such as reduced availability of general practitioners, may also contribute to increased involuntary care and treatment.^23^ Additionally, shortages of mental health professionals can act as barriers to accessing mental healthcare, especially in rural areas.^24^ Regarding resource provision, there has been a decline in resources per person for specialist mental healthcare, while resources for primary health services have increased in recent years.^10^ Societal and clinical factors since 2017 include the COVID-19 pandemic, contributing for instance to a significant rise in eating disorders among adolescents.^25^ Although fewer individuals were diagnosed with substance-induced psychosis during the pandemic in Norway, the total number of such psychotic episodes increased.^26^ Moreover, increased use of more potent substances may have contributed to more substance-induced psychoses.^27^ Despite rising involuntary care rates and acute admissions, as well as more severely ill individuals admitted,^10^ it remains uncertain whether the prevalence of SMIs in Norway has increased since 2017.

### Increased awareness of patient autonomy and improved patient involvement

Our findings indicate that introducing a capacity-based mental health law has contributed to increased awareness of patient autonomy and improved patient involvement. DMC assessments seem to facilitate better communication and closer collaboration. Additionally, more comprehensive assessments, with an increased emphasis on non-legally binding advanced statements, indicate improved participant involvement. Because of the limited number of participants in this study, particularly in the 2022–23 interview round, it is not possible to conclude whether these findings illustrate broader patient experiences. However, our findings are consistent with recent Norwegian studies of people whose CTOs were lifted after the 2017 amendments.^29,30^ Notably, these studies indicated that the introduction of a capacity-based mental health law in Norway was associated with increased awareness among health professionals regarding patient autonomy and involvement. According to these studies, health professionals engaged in more extensive dialogue and assessed individuals’ conditions more frequently to adjust treatment. This required expertise, continuity and close collaboration across the healthcare system.^29,30^ Regardless of rising involuntary care and treatment rates, raised awareness of autonomy arguably has an intrinsic value. However, it remains unclear whether increased awareness of autonomy led to introducing DMC or vice versa, and the level of awareness is challenging to quantify. Moreover, introducing DMC accentuates the difficulties of balancing the principles of patient autonomy and beneficence, especially for individuals with fluctuating DMC. Given the complex interplay among the described post-2017 changes in mental healthcare, more efforts appear to be needed to provide the same level of care for some people as before the amendments. Thus, while increased awareness of autonomy is beneficial, it does not necessarily diminish patient needs.

### Altered thresholds for admission and discharge and increased involuntary care rates

Key findings suggest higher admission thresholds and lower discharge thresholds in mental healthcare after 2017, potentially contributing to people becoming more severely ill. Fewer individuals seem to be admitted voluntarily, while those admitted involuntarily are more severely ill with worse discharge conditions. This aligns with health registry data after 2017, which indicate increased involuntary care rates, both in hospitals and out-patient clinics under CTOs, as well as a rise in referred patients who are not being involuntarily admitted.^10^ Factors such as reduced availability of in-patient beds and health professionals, possibly combined with strict evidentiary requirements and limited training in DMC assessments (see Fig. 1), could contribute to the altered thresholds and increased illness severity. The inadequate implementation of DMC, including a lack of systematic training and validated tools, may also have contributed to this development.^10^ While findings from the 2018 and 2022–23 interviews generally show similarities, the interim involuntary care reduction in 2017 may account for the somewhat different experiences among participants in the two interviews rounds: they had recently experienced this reduction in 2018 and observed a return to a gradual increase along the pre-2017 trajectory by 2022–23. Thus, the complex interplay of factors within a mental healthcare system suggests that individual factors, like DMC, do not operate in isolation.^31^

The rising involuntary care and treatment rates may be reinforced by a pre-2017 legal mandate in Norwegian health law to use formal involuntary care measures when incompetent individuals do not resist care (see Appendix Table 4). This has likely contributed to the almost threefold increase in involuntary treatment decisions between 2016 and 2022.^10^ This appears to be a significant and often overlooked factor contributing to rising involuntary care rates in mental healthcare internationally.^11^ However, many human rights advocates view these formal measures as safeguarding patients’ right to liberty and security (e.g. European Convention on Human Rights, Article 5), adding nuance to rising involuntary care and treatment rates. To address this issue, the Norwegian Ministry of Health and Care Services proposed several legislative amendments to the Norwegian Parliament in November 2024, including allowing care and treatment for incompetent, non-resistant patients without requiring a formal involuntary decision.^32^ It remains to be seen whether these proposed legislative amendments will be enacted into law and what impact they will have on involuntary care and treatment rates.

The increased challenges of providing care to people with comorbid substance misuse post-2017 is supported by trends in health registry data. Such comorbidities tend to exacerbate clinical deterioration among individuals with SMIs, who already face considerable barriers to accessing care.^33^ Health registry data also suggest that patient groups with fluctuating DMC may be more vulnerable to inadequate follow-up between 2015 and 2021.^10^ While the overall increase in ‘revolving door’ patients reported by participants post-2017 does not align with health registry data, it appears to apply to cases involving comorbid substance use.^10^ Moreover, differentiating substance-induced psychoses from psychotic illnesses with comorbid substance use can be challenging.^34^ Thus, some of the challenges surrounding substance-induced psychoses may have been attributed to DMC and the challenging legal issue of fluctuating capacity. Further research is needed to explore potential causes and mediating factors, including the DMC criterion and insufficient training in DMC assessments, or broader factors like increased use of more potent substances.

Similar to the experiences in Norway, there was an increased prevalence of CTOs following the implementation of Queensland's capacity-based Mental Health Act 2016, which aimed to strengthen patient autonomy through least restrictive measures and alternatives to involuntary care.^35,36^ Consistent with our findings, possible explanations for this unintended consequence in the Australian jurisdiction included high demands on public mental health services with limited resources, a lack of voluntary treatment options and inadequate implementation of the legislative reform.^35,36^

Our findings suggest that the impact of a capacity-based mental health law depends on the complex interplay of health, social and legal systems. It is debatable whether the 2017 amendments’ objective of reduced involuntary care and treatment was ever achievable, given the reduction in mental healthcare resources per person in specialist care and a stronger emphasis on formal legal measures for incompetent individuals not resisting care.^10^ Rather than reducing involuntary care and treatment, a more realistic objective could have been for complex health, social and legal systems to adjust to the amendments without significant unintended consequences. While the rising involuntary care and treatment rates were unintended, the interplay of these complex systems suggests that this development was less surprising. Similar to the experiences in Queensland and those in the USA following amendments to mental health laws across several states in the 1960s and 70s, our findings challenge the notion that stricter legal standards alone will reduce involuntary care and treatment rates.^35–37^ In addition to stricter standards, reducing involuntary care seems to demand a well-organised and well-funded mental health system with adequate voluntary treatment options.^38^

### Need for improved collaboration in health services

Our findings indicate that primary health services have assumed greater responsibility for follow-up of more severely ill patients post-2017, particularly those with SMIs. Admitting people to specialist care may also involve substantial resources for primary health services, adding to increased resource usage following this shift. Access to voluntary in-patient care seems constrained by the reduced availability of in-patient beds. Moreover, these developments do not seem to have been accompanied by adequate voluntary out-patient follow-up from specialist or primary health services since 2017,^10,39^ leaving individuals with SMIs particularly vulnerable.^40^ Additionally, national health policies may have contributed to prioritising less severe mental conditions, leading to inadequate access to quality care for these individuals.^41^ The responsibilities for people with SMIs in primary care have been increasing for years.^11^ Despite the increased provision of resources to primary care in recent years, the follow-up of this vulnerable patient group remains challenging because of lacking expertise and resources, particularly in cases of comorbid substance misuse.^10^ Additionally, variations among municipalities could exacerbate patient follow-up.^10^ Thus, the post-2017 challenges appear to be related to inadequate capacity, expertise and collaboration in healthcare services. Moreover, fragmented health laws could contribute to exacerbating the lack of coordinated service provision.^10^ Moving forward, closer collaboration across the health system seems necessary to address the needs of individuals with SMIs.

### Increased family responsibility but unchanged involvement

Key findings include increased family responsibility and continued lack of involvement from health services following the 2017 amendments. A 2023 article, based on interviews conducted in 2019–20 with families of individuals whose CTOs were revoked following the 2017 amendments in a specific region of Norway, found that the amendments had minimal impact on families.^42^ The article suggests that reduced involuntary care and treatment and increased patient autonomy improved family well-being and patient treatment. Discrepancies between these findings and ours may stem from differences in geographical area and sample size. Furthermore, the recruitment of participants and families in the article may have led to a sample that included more well-functioning individuals and healthier families. In this study, families were recruited through national organisations, potentially favouring experiences of inadequate family involvement. However, our relatively large sample size, representing all key stakeholder groups and qualitative follow-up design strengthen our findings.

The unchanged family involvement after 2017 indicates that the health services are failing to recognise the potential contribution of families in mental healthcare. Collaborating with, supporting and guiding families can be crucial in preventing and identifying patient deterioration.^43^ Family involvement seems particularly important in assessing fluctuating DMC, as significant discrepancies between patient and family perspectives can offer valuable insights. Systematic family involvement, preferably combined with pharmacological and individual therapy, is recommended for individuals with SMIs and appears essential for ensuring access to quality care.^44^ However, this support is often lacking for patients and their families.^44^ Moreover, improving the handling of confidentiality issues among health professionals is key to advancing family involvement.^10^ Thus, adequate information, and involvement and collaboration with families are likely prerequisites for a well-functioning capacity-based health system.

### Limitations

The study's qualitative design warrants cautious interpretations and conclusions. Quantitative studies are needed to complement our findings and provide a more comprehensive understanding of their impact. While the interview data do not ground generalised conclusions or causal claims, the study design is suited for generating hypotheses based on the reported experiences and careful interpretations. By emphasising the complexity of developments in Norwegian mental healthcare since 2017, the study highlights the interdependence of contributing factors.

Another limitation is the attrition in participants from 60 in 2018 to 26 in 2022–23, partly because of our inclusion criterion. This 43% follow-up rate in 2022–23 limits the value of the qualitative follow-up design of the study. The differential attrition, particularly among primary care and patient participants, limits insights into the legislative amendments. Recruiting people who had experienced involuntary care and treatment after the legislative amendments further reduced participant numbers. The predominance of clinical participants, particularly in 2022–23, combined with a research group consisting of health professionals, may have gravitated the study's focus towards the clinicians’ perspectives. Moreover, the limited number of participants in different groups across various geographic areas hindered the exploration of geographic variation. Maintaining the same number of participants per group in 2022–23 irrespective of previous inclusion could have prevented the differential attrition.

There were differences in the research groups conducting interviews in 2018 and in 2022–23. This was addressed by assessing the researchers’ impact on validity and maintaining reflexivity to minimise researcher bias.^15^

## Conclusions

Introducing a capacity-based mental health law appears to raise awareness of patient autonomy, but its impact depends on the interplay of complex health, social and legal systems. Since 2017, involuntary care rates have risen despite increased thresholds for admissions. Moreover, primary health services and families seem to face challenges in managing more severely ill patients. Several factors may influence these changes, including the implementation of DMC, legal interpretations, formal measures for the care of non-resistant incompetent patients, reduced availability of in-patient beds and health professionals, inadequate voluntary treatment options and societal developments. Although the rising involuntary care rates were unintended by the amendments’ objectives, the post-2017 changes seem less surprising when considering the interplay of these complex systems. Further research is needed to better understand these changes and their causes.

## Supporting information

Jorem et al. supplementary material Jorem et al. supplementary material

## Figures and Tables

**Fig. 2:**
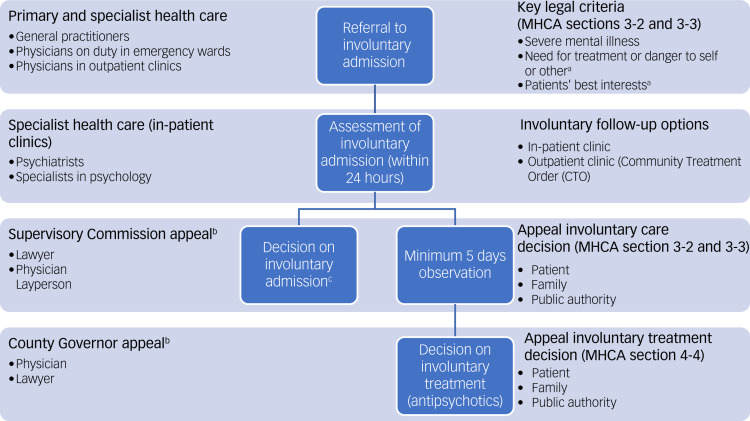
Overview of involuntary care and treatment in accordance with the Norwegian Mental Health Care Act (MHCA). ^a^The danger criterion involves the patient posing a danger to the life or health of self or others without involuntary care. The capacity criterion does not apply in cases of danger except to the patient's own health. If competent patients pose a danger only to their own health, they cannot be involuntarily admitted or treated. For further information, refer to the University of Oslo's unofficial translation of the MHCA, last updated in November 2007.^45^ ^b^Patients, their family or a public authority can appeal involuntary care decisions to the local Control Commission (Kontrollkommisjonen), which consists of a lawyer, physician and layperson. Decisions regarding involuntary treatment can be appealed by the same persons to the County Governor (Statsforvalteren), where a physician and lawyer assess whether the decision is in accordance with MHCA. Appeals must first be made to these local supervisory bodies before the decisions can be taken to the District Courts. ^c^Involuntary care and treatment decisions must be reassessed at least every 3 months.

**Fig. 3:**
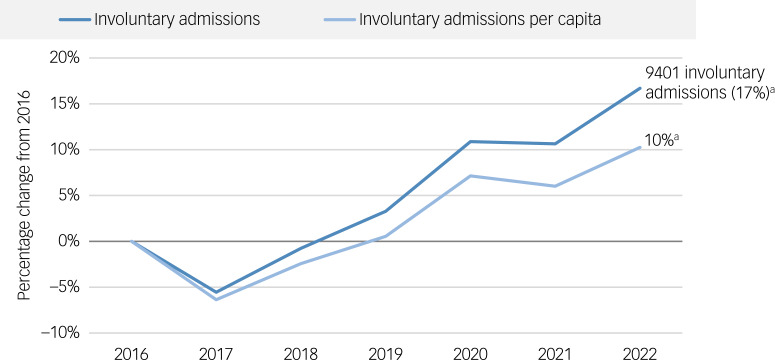
Percentage change in involuntary admissions from 2016 to 2022 (reprinted with permission from the Commission on Decision-Making Capacity, June 2023). ^a^National health registry data reveal that there were 9401 involuntary admissions distributed among 6700 patients in mental healthcare in 2022, representing a 17% increase in involuntary admissions compared to 2016, a 10% increase per capita.^10^

## Data Availability

The data used and analysed during this study are not publicly available to protect the privacy of participants, in compliance with approvals from the Data Protection Officer at the University of Oslo and the Norwegian Agency for Shared Services in Education and Research. Access to the data has been restricted to study personnel, and all data are securely stored on the University of Oslo's services for sensitive data. Data are provided within the manuscript.

## Appendix

**Table 3:** Relevant sections with their corresponding wording in Norwegian health legislation related to DMC assessments in mental healthcare. For further information, refer to the University of Oslo's unofficial translation of the MHCA, last updated in November 2007^45^

Paragraph	Wording
Patients’ and Users’ Rights Act section 4–3, paragraph 2	‘Competence to consent may be wholly or partly suspended if the patient, due to a physical or mental disorder, senile dementia or mental retardation, is *obviously* unable to understand what the consent entails.’
Mental Health Care Act section 3–3, paragraph 4^a^	‘The patient lacks competence to consent, cf. the Patient and Users’ Rights Act section 4-3. This criterion does not apply when the person poses an obvious and serious risk to their own life or the life or health of others.’

a. The wording in the Mental Health Care Act (MHCA) section 3–2, paragraph 3 regarding involuntary observation and MHCA section 4–4, paragraph 1 regarding involuntary treatment is identical to that in MHCA section 3–3, paragraph 4 (own translation).

**Table 4:** The comprehensive revision of MHCA in 2017 entailed procedural rights to ensure patient autonomy^11^

Amendments to the Mental Health Care Act (MHCA) in 2017
Altered statutory objectives to prevent and limit the use of involuntary mental healthcare and to strengthen patient autonomy (MHCA section 1–1)
The right to free legal assistance was expanded to cover complaints about decisions on involuntary treatment (MHCA section 1–7)
More strict requirements for justification of decisions requiring increased documentation (MHCA sections 3–3 a and 4–4 a)
The right for patients and family to express their views and experiences on involuntary care and treatment before a decision is made (MHCA sections 3–3 a, 4–2 and 4–4 a)
The mandatory observation period was extended from three to five days before a decision on involuntary treatment can be made (MHCA sections 4–4 and 4–4 a)
Stronger emphasis on the existing legal mandate to use formal involuntary care and treatment measures when incompetent patients did not resist admission or treatment (Patient and Users’ Rights Act section 4–3)

**Table 5:** Themes, subthemes and illustrative quotes from 2022–23 regarding the impact of introducing a capacity-based mental health law in Norway in 2017 (see Table 2 for additional quotes)

Theme	Subtheme	Quote (type of participant, participant number, quotation number)
**1. Increased awareness of patient autonomy and improved patient involvement**	1A. Increased awareness	‘If one manages to assess DMC reasonably accurately, it is a right that strengthens the legal protection for competent patients who should not be subjected to anything they do not consent to. However, this is somewhat overshadowed by the difficulty of making these assessments.’ (Psychiatrist, 3, Q15)
	1B. Improved patient involvement	‘Perhaps we were more thorough, and the conversations were better in the beginning regarding involving the patient in the decision-making process. Over time, however, a combination of fatigue and limited time may have led to a more lenient approach. It is possible that the legislative amendments initially had an instructive effect and improved practices, but over time, this may have slipped somewhat.’ (Psychiatrist, 10, Q16)
**2. Altered admission and discharge thresholds and challenges in helping patient groups**	2A. Increased admission thresholds and reduced discharge thresholds	‘I feel that we consistently experience readmissions. It is a recurring pattern: the patient regaining capacity, terminating involuntary care, then returning after four months. It seems to happen frequently. However, I believe the management has indicated that the figures do not differ significantly. There is also a subjective factor here, a desire for things to be this way. So, every time we observe a patient terminating involuntary admission and medication in May, only to be readmitted in August, we think, “This is due to capacity.” It becomes a self-reinforcing negativity that may not align with reality.’ (Psychiatrist, 1, Q17)
	2B. More challenging to help certain patient groups	‘This concerns the most vulnerable patients, suffering from severe mental illness, often with comorbid substance misuse. I find that they frequently fall through the cracks because they regain capacity, shortening the duration of involuntary admission.’ (Psychiatrist, 2)
		‘Our concern regarding patients with manic symptoms has been particularly pronounced in light of the legislative amendments. I often find that the threshold for admission has been significantly raised, leading to many unnecessary rounds before finally being admitted. Additionally, they are often discharged prematurely, resulting in relapses. Instead of the expected 4to 6weeks, the recovery period may extend to several months or even half a year. This may have detrimental effects on these patients’ finances and relationships.’ (Psychiatrist, 7, Q18)
**3. More responsibility for primary health services with inadequate assessment training**		‘There has been a lack of training, particularly in primary healthcare services. They were quite unprepared for the legislative amendments.’ (Psychiatrist 3, Q19)
**4. Increased family responsibility but unchanged involvement by health services**	4A. Increased responsibility	‘I believe it has been more burdensome for families since 2017.’ (Psychiatrist, 3)
		‘Ensure readmissions, call the police, call an ambulance – these are the responsibilities of families. They have been assigned greater responsibility; indeed, they have.’ (Psychiatrist, 1, Q20)
	4B. Unchanged involvement by health services	‘We rely on families to gather information about the patient. We inform them, in accordance with the law, that they have the right to appeal. However, in my experience, family members are not particularly concerned with decision-making capacity. [...] I feel that I have always spent time informing family members, gathering background information about the patient, and involving them, as they are so important in the treatment process – though perhaps more so after a hospital admission. At the same time, we must also protect families.’ (Psychiatrist, 3, Q21)
