# Supplementary material for: Impact of introducing a capacity-based mental health law in Norway: qualitative exploration of multi-stakeholder perspectives
Source: BJPsych Open. 2025 Feb 25;11(2):e35. doi: 10.1192/bjo.2024.810 (PMC12001955; doi:10.1192/bjo.2024.810)
Supplement: Jorem et al. supplementary material [file S205647242400810Xsup001.pdf]

Supplementary Material 1: Interview Guide

Aims: Assess the impact of the legislative amendments, the quality of assessments of decision-making capacity (DMC), and how they are conducted in the context of involuntary observation and care, Community Treatment Orders, and involuntary treatment. The term "quality" refers to whether the DMC assessments are good and accurate (validity).

Questions

1. *What are your experiences with the impact of introducing DMC as part of the amendments to the Mental Health Care Act in 2017?*

- For example, what consequences (positive and negative) has the DMC criterion had?
- For example, increased autonomy and dialogue, or whether the DMC assessment is perceived as demeaning?
- Reduced involuntary care (if so, what types)?
- Consequences for informal involuntary care, patient deterioration, readmissions, time spent (documentation, dialogue, follow-up), requirements for collaboration, burden on families?

2. *Do you have other experiences of how DMC assessments are conducted after the legislative amendments?*

3. *How is DMC assessed? How do you evaluate the quality of the assessments? Are there any challenges related to these assessments? Do you find that DMC is assessed similarly?*

4. *What is the need for training and tools to assess DMC?*

5. *How have the legislative amendments influenced your practice in other areas of health law?*

- For example, in cases of doubt regarding DMC, is involuntary observation used?
- Emphasis on the patient's wishes and experiences, altered use of the danger criterion?
- Is DMC assessed when patients collaborate, or families are involved?
